# Paroxysmal sympathetic hyperactivity in brainstem-compressing huge benign tumors: clinical experiences and literature review

**DOI:** 10.1186/s40064-016-1898-x

**Published:** 2016-03-16

**Authors:** Seungjoo Lee, Go Woon Jun, Sang Beom Jeon, Chang Jin Kim, Jeong Hoon Kim

**Affiliations:** Department of Neurological Surgery, Asan Medical Center, College of Medicine, University of Ulsan, 388-1 Pungnab-dong, Songpa-gu, Seoul, 138-736 Republic of Korea; Department of Anesthesia, Bestian Medical Center, Daejeon City, 300-060 Republic of Korea; Department of Intensive Care Medicine, Asan Medical Center, College of Medicine, University of Ulsan, Seoul, 138-736 Republic of Korea

**Keywords:** Paroxysmal sympathetic hyperactivity, Brain tumor, Brainstem, Opioid

## Abstract

Severe paroxysmal sympathetic overactivity occurs in a subgroup of patients with acquired brain injuries including traumatic brain injury, hypoxia, infection and tumor-related complications. This condition is characterized by sudden increase of heart rate, respiratory rate, blood pressure, body temperature and excessive diaphoresis. The episodes may be induced by external stimulation or may occur spontaneously. Frequent occurrence of this condition could result in secondary morbidities, therefore, should be diagnosed and managed insightfully. These symptoms could be confused with seizures or other medical conditions, leading to unnecessary treatment. Despite clinical significance of paroxysmal sympathetic hyperactivity (PSH), brain tumor-induced PSH has not been studied nearly. In this report, two cases of the PSH in patients with brainstem-compressing benign tumors were introduced. The most useful pharmacologic agents were opioid (e.g., fentanyl patch) in preventing PSH attack, and nonselective β-blocker (e.g., propranolol) in relieving the symptoms. Clinical experiences of the rare cases of benign tumor-induced PSH can be helpful as an essential basis for further research.

## Background

Episodes of acute sympathetic disturbances may occur after acquired brain injuries, such as traumatic brain injury, hypoxia, infection, and tumor-related complications (Baguley et al. 2004; Baguley et al. 2007). This condition could be misinterpreted as seizure, sepsis, metabolic disturbances because of clinical similarities with them. In patients with acquired brain injury, a paroxysmal sympathetic hyperactivity (PSH) characterized by sudden increase of heart rate, respiratory rate, blood pressure, body temperature, diaphoresis as well as extensor posturing could be manifested (Meyer 2014), and had been termed as dysautonomia, diencephalic epilepsy, sympathetic storming, and autonomic dysfunction syndrome (Goddeau et al. 2007; Rabinstein and Benarroch 2008). Recently, a PSH has been used to describe the above conditions (Meyer 2014). Although the pathophysiology of the PSH is still elusive, the acquired brain injuries involving brainstem have been revealed to cause the PSH (Follett et al. 2003; Soukup et al. 2002). The frequent episodes of PSH could result in secondary morbidities including increased intracranial pressure, cardiac damage, and metabolic disturbances. Therefore, this condition should be diagnosed and managed insightfully (Reith et al. 1996; Thorley et al. 2001).

In this report, the rare cases of postoperative PSH in huge benign brain tumors compressing brainstem would be introduced, in addition, the clinical experiences regarding medical treatment of PSH would be also presented.

## Case reports

### Case 1

**Fig. 1:**
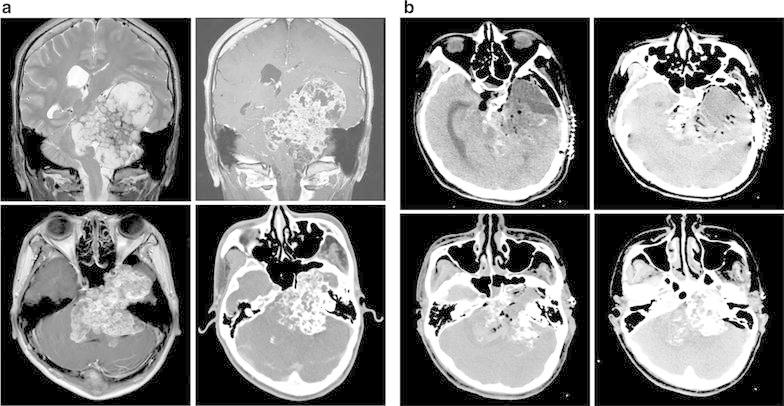
**a**. Preoperative radiologic imaging of patient 1 with PSH. Huge size (8.1 cm) extraaxial calcified mass over left middle cranial fossa and posterior cranial fossa is compressing brainstem. This mass originates from petrosal apex and displaces midbrain, pons, and middle cerebellar peduncle to right side. (*Clockwise direction*; T2WI coronal, T1W enhance coronal, CT with enhance, T1W enhance axial). **b**. Postoperative (POD 20) CT scan showing residual calcified mass adjacent to brainstem and hemorrhage, edema in brainstem

**Fig. 2:**
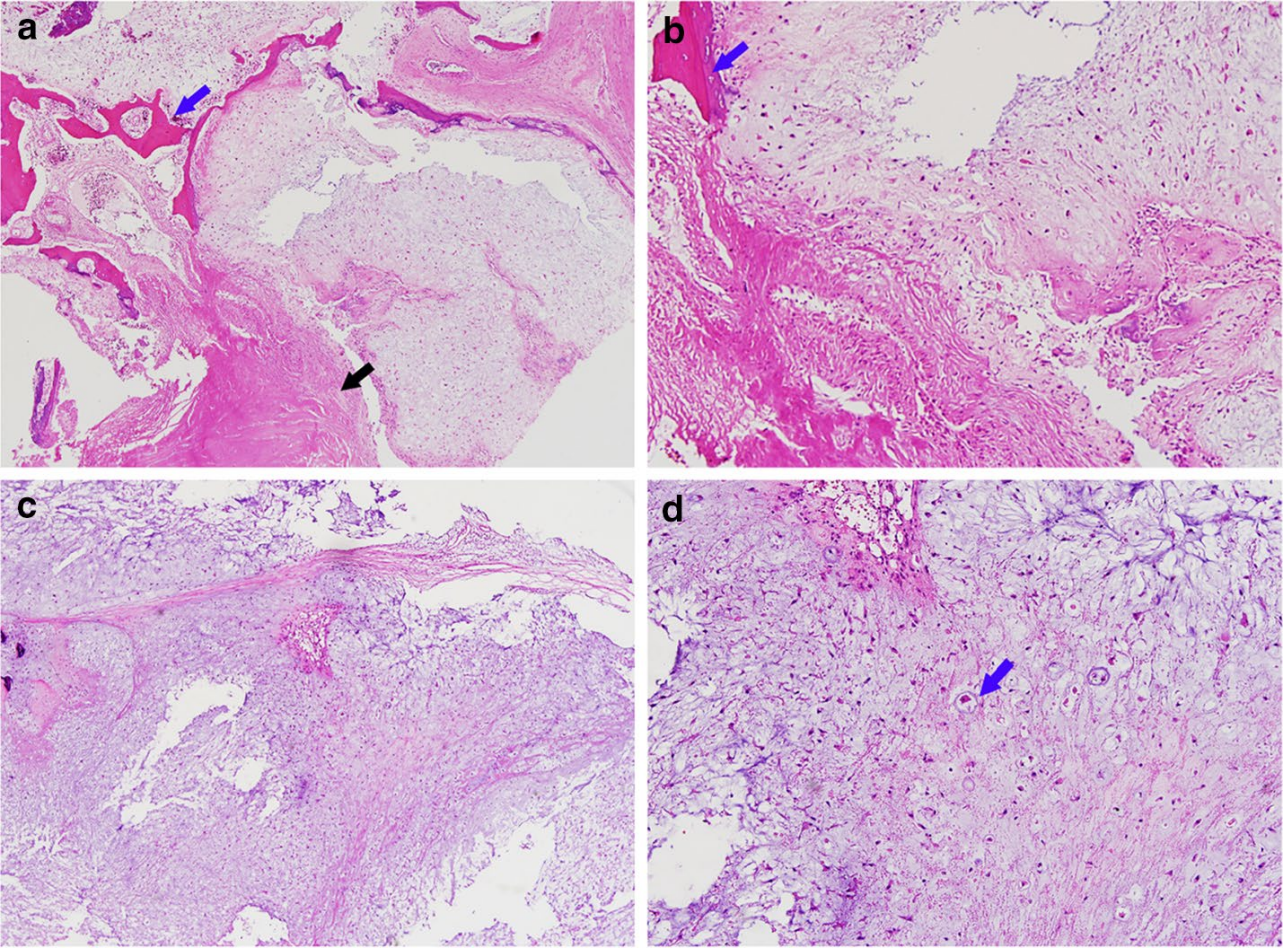
Histopathology of patient 1’s specimen (hematoxylin and eosin stain). Distinctly, there are abundant fibrous and chondroid matrix with formation of pink homogenous osteoid (*Blue arrow*, in **a**, **b**). Some areas of the tumor show obvious cartilaginous differentiation with well-formed hyaline cartilage (*black arrow* in **a**). Most areas of the tumor consist of cellular proliferation of small rounded chondrocytes (*blue arrow* in **d**). These cells are focally arranged in cords and trabeculae (**c**). Based on these findings, patient was diagnosed chondrosarcoma. (**a**, **c**: 40×, **b**, **d**: 100×)

A 23-year old man was referred with symptoms of progressing left side hemiparesis (grade II) and intermittent dystonic movements. In neurologic examination, he demonstrated alert mentality and his pupil was slightly dilated and decreased light reflex in right side. In addition, it is also identified that the decreased swallowing function and dysarthria. A MRI scan showed an 8.1 cm-sized calcified extraaxial mass over left middle and posterior cranial fossa with petrosal apex epicenter. At the same time, the midbrain, pons, and left middle cerebral peduncle was compressed and deviated to right side (Fig. 1a). The patient underwent the surgery via the left transzygomatic subtemporal transtentorial approach and the tumor was subtotally removed due to the firm adhesion of tumor to the brainstem. In the postoperative CT scan, focal hemorrhage and diffuse edematous change were observed in middle cerebral peduncle and brainstem (Fig. 1b). The level of consciousness of patient was stupor and sustained thereafter. The histopathologic diagnosis of this patient was chondrosarcoma consisting of cellular proliferation of small rounded chondrocytes and obvious well-formed hyaline cartilage (Fig. 2a–d).

**Fig. 3:**
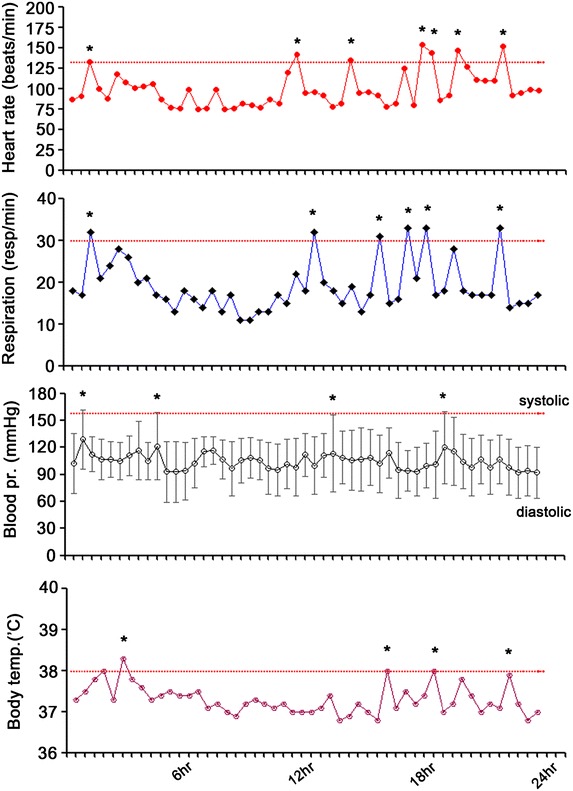
Vital signs of patients with PSH attack. *Asterisks* (*) indicate outlier points above normal limit. Gradations on *X axis* indicate 30 min intervals

At 23 days after surgery, it was often showed that the paroxysmal increase of heart rate, respiratory rate, blood pressure and extensor posturing responding to non-noxious stimuli such as bathing and turning of body. These symptoms were repeated and lasted for several minutes and then, spontaneously subsided. This over-reactivity of sympathetic responses to external stimuli was manifested as the increase of respiratory rate, heart rate, blood pressure and body temperature even diaphoresis (Fig. 3). The PSH was occurred suddenly, a literal meaning. The symptoms were onset response to non-noxious stimuli such as head turning, chest percussion or presented even without stimuli. The PSH was usually sustained within 10–20 min and resolved spontaneously. These wax and wine pattern was repeated several times a day.

To exclude postoperative seizure, the electroencephalogram was continuously monitored, however, the epileptiform discharges were not observed. Moreover, in spite of antiepileptic drugs (AEDs) were administrated and maintained within therapeutic level of concentration in blood, the paroxysmal sympathetic hyperactivities (PSH) were repeated. In blood chemistry, the level of glucose and electrolytes were within normal ranges. The inflammation markers, such as WBC, ESR, CRP, and lactic acid were also not significant. There were no active lung lesions on chest X-ray and urine analysis was also normal.

Initially, the beta antagonist (propranolol, 1 mg/kg/day) and diazepam (2 mg/day) were tried under impression of PSH for several days, however, there was no effect in prevention of the PSH. The baclofen (10 mg/tid/day) was added, but not effective. The opioid patch was applied and the occurrence of the PSH was gradually decreased over a week. After 10 days of the opioid patch application, the PSH was not observed, however, abdominal distention and constipation occurred. To relief the constipation, the opioid patch was removed, and then the PSH was recurred at 3 days after cessation of the patch. Therefore, the opioid patch (fentanyl propanamide, 25 mcg/hr) was reapplied and PSH was subsided again. At same time, stool softner (magnesium oxide, 500 mg/tid/day) and bowel motility drugs (domperidone, 10 mg/tid/day) were administered and intermittent enema was also performed. The use of the opioid patch was tapered gradually after 1 week of reapplication, and the PSH was not recurred.

### Case 2

**Fig. 4:**
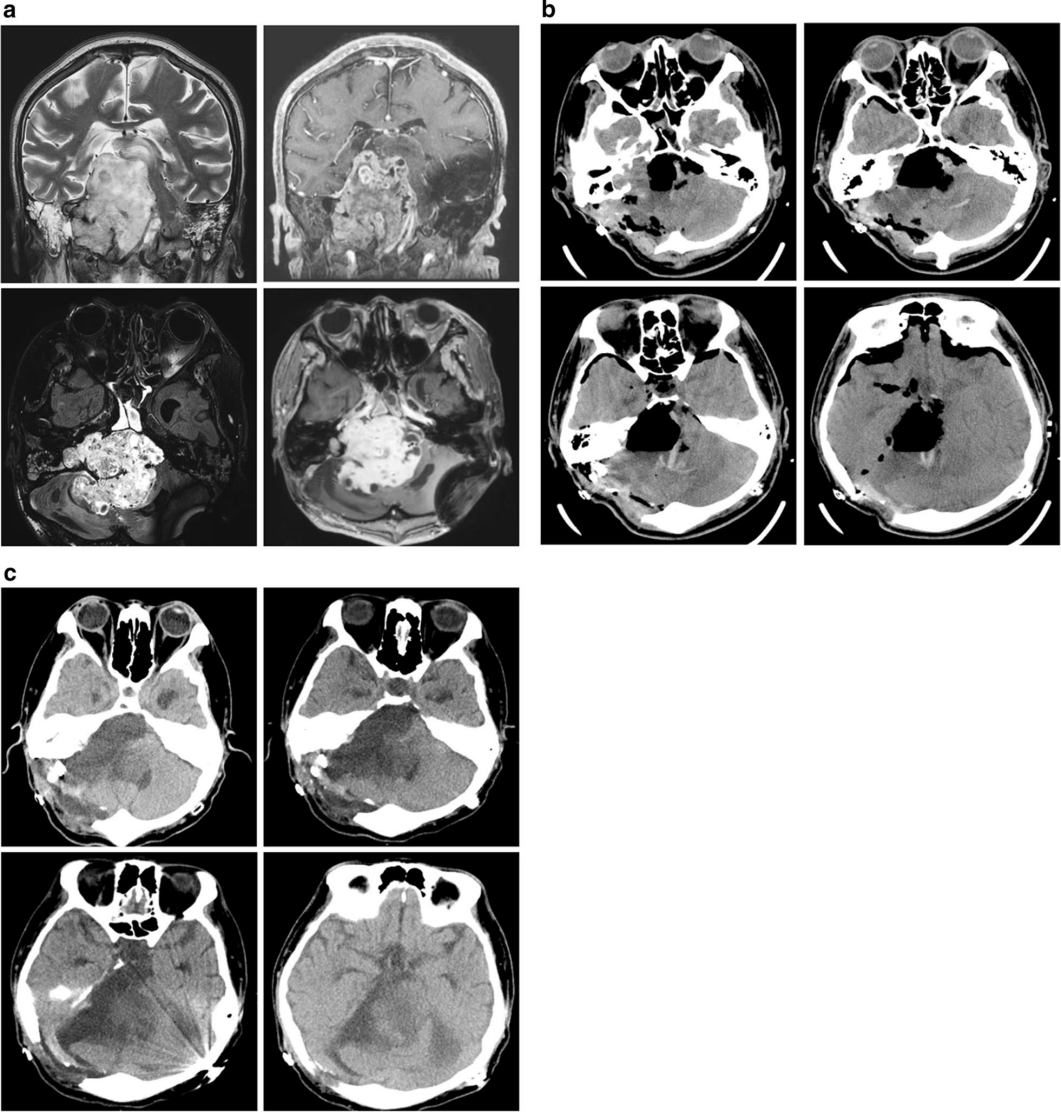
**a**. Preoperative MRI of patient 2 showing PSH. A 7.7 cm-sized homogenous enhancing cystic and solid extraaxial mass originates form jugular foramen. This mass extends from retroclival area to posterior fossa compressing brainstem. (*Clockwise direction*; T2WI coronal, T1W enhance coronal, T1 W enhance axial, 3D FLAIR). **b**. Immediate postoperative CT scan demonstrating focal hemorrhage and edema in compressed brainstem. **c**. Postoperative CT scan (POD 14) when the PSH occurred

A 30-year-old man was referred with stupor consciousness from other hospital after primary surgery. He was diagnosed as the huge sized jugular foramen schwannoma with compressing brainstem (Fig. 4a). He already underwent the ventriculoperitoneal shunt to relief obstructive hydrocephalus and suboccipital craniotomy with partial decompression of the tumor. However, the residual mass was still huge and compressing the brainstem. He had been stupor with decreased brainstem reflex, such as light pupillary reflex, corneal and gag reflex.

**Fig. 5:**
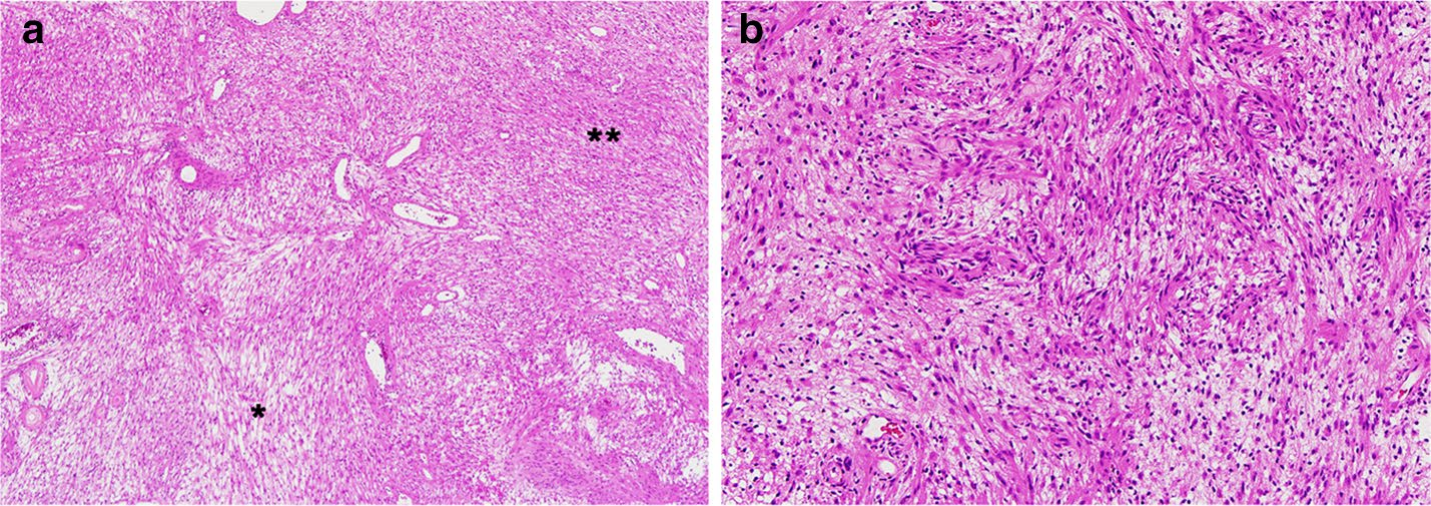
Histopathology of patient 2’s specimen (H&E stain). **a**. Biphasic pattern with cellar Antoni A (*single asterisk*) and hypocellular Antoni B (*dual asterisk*) areas. **b**. Schwann cell nuclei forming palisades focally and showing compact fasciles of elongated tumor cells with nuclear polymorphism. Based on these findings, patient was diagnosed schwannoma. (**a**: 40×, **b**: 100×)

The mass was subtotally removed via previous craniotomy site. The firmly adhesion lesion to brainstem was left, because of concerning about brainstem injury. In postoperative CT scan, it was observed the focal hemorrhage and mild edema in brainstem (Fig. 4b). His neurological status was not changed and maintained unrelievedly. In histopathology of this patient, the tumor composed entirely of neoplastic Schwann cells and forming two basic patterns in varying proportion: areas of compact, elongated cells with occasional nuclear palisading (Antoni A pattern) and less cellular, loosely textured cells with variable lipidization (Antoni B pattern). Based on these findings, this patient was diagnosed schwannoma (Fig. 5).

At 2 weeks after surgery, the paroxysmal increase of the heart rate, respiratory rate and blood pressure was observed, as well as extensor posturing, reactive to bathing or repositioning of the body. These symptoms were resolved spontaneously within several minutes. There were no significant findings in EEG, CT scan, and blood chemistry. The diazepam and baclofen were not effective in preventing the PSH, therefore, the opioid patch (fentanyl propanamide, 25 mcg/hr) was applied. The PSH had been disappeared gradually over a week. The opioid patch was used totally for 2 weeks, the PSH didn’t recur any more.

## Discussion

### Diagnosis and nomenclature

The distinctive clinical features of sudden increase of sympathetic activities responding to non-noxious external stimuli have been described a wide range of labels, dysautonomia, sympathetic storming, hypothalamic-midbrain dysregulation syndrome, diencephalic epilepsy, and paroxysmal sympathetic hyperactivity (PSH) (Rabinstein 2004). Recent systemic reviews about this phenomena have been converged to the PSH because of well-delineation of clinical features such as increasing of heart rate (HR), respiratory rate (RR), blood pressure (BP), body temperature (BT), diaphoresis and extensor muscle tones (Table 1). In some cases, combinations of sympathetic and parasympathetic overactivity as well as pure sympathetic overactivity were observed, therefore, sometimes separated into two categories: pure PSH (i.e., absence of parasympathetic features) and mixed autonomic hyperactivity (i.e., combinations of sympathetic and parasympathetic overactivity) (Meyer 2014). The cases in this report are considered to the pure PSH. Clinical manifestations of cases in this reports were paroxysmal HR of 180 beats/min, RR of 40 breaths/min, BP of 156 mmHg, neurogenic hyperthermia, and decerebrate posturing. The paroxysms were precipitated by afferent stimulation, a feature that has recently been confirmed in systemic review about PSH (Rabinstein 2004; Rabinstein 2007).

**Table 1:** Diagnostic criteria of PSH: transient presence of four of the six criteria without other potential causes

	Clinical features
Fever	Body temperature > 38.3 °C
Tachycardia	Heart rate > 120 beats/min or >100 beats/min with β-blocker
Hypertension	Systolic blood pressure > 160 mmHg or pulse pressure > 80 mmHg
Tachypnea	Respiratory rate > 30 breaths/min
Excessive diaphoresis	
Extensor posturing or severe dystonia	

### Clinical relevance

PSH is an important clinical problem encountering in critical care or rehabilitation setting of severely brain injured patients. The retrospective studies about prognosis of patients with the PSH revealed that the clinical outcomes measured by Glasgow outcome scale (GOS) (Goh et al. 1999), functional independence measure, and hospital length of stay (LOS) were poorer in patients with the PSH (Baguley et al. 2009). Excessive sympathetic nervous system activation causes of increased potential secondary morbidities (Baguley et al. 2008; Pranzatelli et al. 1991). Hypermetabolic state during sympathetic storms leads to body weight decrease estimated at 25 % in the acute period alone (Lemke 2007). These patients also have an increased likelihood of developing hyperthermia has been found to predict poor outcome, potentially as a direct cause of secondary brain damage (Rabinstein and Benarroch 2008; Soriano et al. 2014). Moreover, the prolonged hypersympathetic tone associated with the PSH could produce or exacerbate cardiac damage and elevated intracranial pressure (Rodriguez et al. 2006; Tong et al. 2000). In this report, it was observed the 5 kg of body weight decrease during a week associated with the PSH. Muscle enzymes were also readily elevated after PSH attack.

### Etiology, pathophysiology, and differential diagnosis

Several types of acquired brain injury preceding PSH occurrence were reviewed. The PSH was the most commonly associated with traumatic brain injury (79.4 %), followed by hypoxia (9.7 %), stroke (5.4 %), unspecified (1.8 %), hydrocephalus (2.6 %), tumor (0.6 %), hypoglycemia (0.3 %), CNS infection (0.3 %) in precedence factors order (Meyer 2014). Although the pathophysiology of PSH has not been fully investigated, recently brainstem disconnection theory has been spotlighted (Baguley et al. 2004). In the earliest articles of PSH, this condition was regarded as an epileptiform manifestation in nature after insults by brainstem involved lesion (Baguley et al. 2007; Sandel et al. 1986). However, the studies that subsequently attempted to identify epileptiform discharges in PSH using electroencephalography demonstrated negative results (Gandhavadi 1988; Kao et al. 2003; Mehta et al. 2008), and most antiepileptic drugs were reported to be ineffective in controlling the condition. As a result of these observations, most researchers have proposed a disconnection theory. This theory assumed that brainstem excitatory centers are released form higher control with a consequent sympathetic hyperactivity (Meyer 2014). Instead, another disconnection theory, the excitatory-inhibitory radio model also have been posed. In this theory, the causative brainstem centers are inhibitory in nature, and sympathetic hyperactivity originates at the spinal cord level analogous to autonomic dysreflexia following high thoracic spinal cord injury. Most previous studies about PSH was observed in traumatic brain injury (79.4 %) or hypoxia (9.7 %). Only 5 cases of PSH were reported in tumor-related conditions, even they were directly midbrain involved gliomas (Goddeau et al. 2007; Meyer 2014; Perkes et al. 2010). In the case of tumors involving diencephalon, the pathophysiology is most likely an activation or a disinhibition of the central sympathetic regions, such as the paraventricular hypothalamic nuclei, lateral periaqueductal gray substance, or rostral ventrolateral medulla. Sympathetic excitation has been demonstrated to occur in experimental neurogenic hypertension in which there are lesions in the hypothalamus or medulla. Moreover, the activation of sympathetic spinal or medullary reflexes, which are triggered from muscle mechanoreceptors and chemoreceptors during episodes of hypertonia, likely have a contributory role as well. It is important, therefore, to recognize the clinical features of these autonomic episodes and to treat them in an appropriate fashion. In this patient the sympathetic storms necessitated some form of blockade or suppression.

Until now, the postoperative PSH in brainstem compression benign tumors were not extensively reported. In this report, huge petroclival chondrosarcoma (patient 1) and jugular foramen schwannoma (patient 2) were compressing brainstem with edematous changes. In both cases, the tumors were firmly adherent to brainstem, as a result, some of tumors remained at brainstem region. Focal brainstem hemorrhage and edema were observed at brainstem, although, the neurologic status was not changed relative to preoperative condition. After 3–4 weeks after surgery, a paroxysmal sympathetic hyperactivities, increasing HR, RR, BP, BT and extensor posturing were observed responding to non-noxious stimuli. These clinical phenotypes were needed to make a differential diagnosis from several clinical conditions including seizure, hydrocephalus, encephalitis, electrolyte imbalance, hypoglycemia, sepsis, and spinal cord injury. Initially, EEG was monitored to exclude seizure, however, no epileptiform discharges were identified. In blood chemistry, electrolytes, glucose, CO2 levels were within normal range, and there were no evidence for inflammatory condition. A CT scan also showed no hydrocephalus. Numerous researches have proposed lesion locations associated with the PSH, however, the definite lesions induced the PSH were still elusive.

Characteristically, a number of previous studies have reported an association between afferent stimuli and sympathetic paroxysms (Bhigjee et al. 1985; Meyer 2014; Ryan et al. 2003; Soukup et al. 2002). Both noxious and non-noxious stimuli such as bathing, body positioning and tracheostomy care invoked a paroxysmal sympathetic hyperactivity. The cumulative evidences from earlier studies support the contention that over-reactivity to afferent stimuli might be the hallmark of the PSH (Baguley et al. 1999). In our cases, the PSH was invoked by non-noxious stimuli such as chest percussion, wound dressing, and bathing. Although, the PSH subsided over 20–30 min spontaneously, so frequently recurred that it could not help going untreated.

### Management

Although there is a historical lack of standardized outcome measures to evaluate treatment efficacy, previous studies used qualitative assessment to describe the effect of intervention on clinical parameters such as fewer paroxysms or paroxysms of reduced intensity (Baguley et al. 2006; Boeve et al. 1998). Several clinical trials about controlling the PSH showed no effect, which have included hydralazine (Lemke 2007), hydroxyzine, diphenhydramine (Dolce et al. 2008), trihexyphenidyl, and proprantheline. On the other hand, sodium amytal, propranolol (Rabinstein 2007), thorazine (Thorley et al. 2001), botulinum toxin A (Jennett et al. 1981), codeine (Cuny et al. 2001), dexmedotomidine (Gandhavadi 1988), prazocin (Follett et al. 2003), and oxycodone (Srinivasan et al. 2007) have demonstrated a beneficial effect on the PSH. The case reports about intrathecal baclofen have proved an effect on the PSH, consistently. However, this intervention is costly, invasive, not always possible (Dooling and Richardson 1976). In our cases, prazocin, propranolol, oral baclofen, benzodiazepine, and opioid were used to relieve the PSH. In terms of frequency and intensity of the PSH, prazocin, baclofen, and benzodiazepine showed little effect. The propranolol, β-antagonist had limited effects to lower the BP, HR on acute paroxysmal situation, but there was no effect to prevent the attack of PSH. The most effectual medication was opioid patch, in case of intolerable condition via per os. Above all, the frequency of the PSH was decreased on applying the opioid patch. The PSH had gradually subsided over a week. Despite this impressive results of opioid patch, constipation was a troublesome side effect after 2 weeks of using the patch. On tapering of the opioid patch, the PSH was recurred, therefore the patch was maintained with intermittent bowel enema and bowel motility drugs (Table 2).

**Table 2:** Summary of commonly used pharmacologic agents in PSH

Drug	Dose	Mechanism	Adverse effect	Our experience	Special points
Opioids	2–8 mg (IV morphine) 10–30 mcg/hr (fentanyl patch)	Reducing pain response, blunting sympathetic activity	Respiratory depression, constipation, ileus hypotension over-sedation	Most effective in preventing PSH event	Need adequate dose titration
Bromocriptine	1.25 mg/bid/day (PO)	D_2_ agonist	Confusion, dyskinesia, hypotension	Not used	Titrate up to 10–40 mg/day
Baclofen	5 mg/tid/day (PO)	GABA_B_ agonist	Muscle weakness, sedation, liver enzyme elevation, bronchial hyperactivity	Not effective	PO agent is not effective
Propranolol	20–60 mg/qid/day (PO)	Nonselective β-blocker	Negative inotropic effect, bronchospasm, hypoglycemia	Effective in decreasing BP and HR during PSH attack	Contraindication in COPD, AV block, Heart failure
Benzodiazepines	Midazolam 1–2 mg IV Lorazepam 2–4 mg IV Diazepam 5–10 mg IV	GABA_A_ agonist	Respiratory depression, hypotension, over-sedation	Not effective	Diazepam may be preferred in PSH
Dantrolene	0.25–2 mg/kg/bid/Day (IV)	Muscle excitation–contraction dissociation by blocking Ca2 + release from sarcoplasmic reticulum	Fatal hepatotoxicity, respiratory depression	Not used	Effective for amelioration of dystonic posturing
Gabapentin	300–900 mg/day (PO)	Block α2δ subunit of voltage-gated Ca2 + channel, inhibiting neurotransmitter release in CNS	Sedation, lethargy	Not used	Titrate up to 3600–4800 mg/day

In addition to pharmacological intervention, there also appears to be a role for nutritional support relating to the caloric energy and replacing the insensible fluid losses associated with higher metabolic rate and diaphoresis (Baguley et al. 2007).

### Natural course

The incidence of the PSH ranges from 7.7 to 33 % of patients with moderate or severe TBI (Baguley et al. 2009; Diamond et al. 2005; Rabinstein 2004). Hypoxic brain injury was the precedent etiology in 9.7–29 % of PSH cases (Reith et al. 1996), and a significant contributor to PSH. However, the greater incidence of TBI gives a higher overall prevalence of PSH. Most cases of the PSH reported in the literature focused on the post-TBI or hypoxia, consequently the natural course of the PSH in postoperative tumor condition was unknown. In severe TBI, only a small proportion of PSH cases (7 %) achieved a moderate or good recovery (Baguley et al. 2004; Rabinstein and Benarroch 2008). In our cases, the tumor-induced PSH was resolved within 2 months with medical treatment. It is not clear whether the relief of the PSH in our clinical setting was obtained by the medical management or the spontaneous resolution of focal hemorrhage and edema in brainstem. At immediate postoperative period, the patient 1 was stupor and sustained during 1 month. However, the patient was recovered gradually and now simple obey-command exam is possible and have a rehabilitation at 5 months after surgery. The case of patient 2 was also stupor at time of surgery and sustained during immediate postoperative period. However, the patient was possible simple obey-command exam, now at 3 month after surgery.

## Conclusions

A variety of acquired brain injuries such as traumatic brain injury (TBI) and hypoxia could cause PSH. Although the pathophysiology of the PSH is still elusive, it appears to be evident that brainstem injury is associated with the PSH. Most cases of the PSH reported to date occurred after TBI and hypoxia. However, the postoperative PSH cases in benign brain tumors compressing brainstem have not been reported extensively. In this report, the postoperative PSH in huge benign brain tumors compressing brainstem was effectively treated with opioid patch. The opioid was effective in preventing the PSH spell, as well as the intensity of it. The constipation was occasionally problematic, however, it could be controlled with intermittent enema and bowel motility drugs.

## Patient consent

The patient and guardian have consented to the submission of the case report for submission to the SpringerPlus.

## Abbreviations

AEDs: antiepileptic drugs
CT: computed tomography
HR: heart rate
RR: respiratory rate
BP: blood pressure
BT: body temperature
PSH: paroxysmal sympathetic hyperactivity
